# Analysis of *TSG101* tumour susceptibility gene transcripts in cervical and endometrial cancers

**DOI:** 10.1038/sj.bjc.6690069

**Published:** 1999-02

**Authors:** J-G Chang, T-H Su, H-J Wei, J-C Wang, Y-J Chen, C-P Chang, C-J Jeng

**Affiliations:** Department of Medical Research, Division of Molecular Medicine, Mackay Memorial Hospital, Taipei, Taiwan; Department of Molecular Medicine, Taipei Municipal Jen-Ai Hospital, Taipei, Taiwan; Department of Medical Research, China Medical College Hospital, Taichung, Taiwan; Department of Obstetrics and Gynecology, Mackay Memorial Hospital, Taipei, Taiwan; Department of Obstetrics and Gynecology, Taipei Medical College Hospital, Taipei, Taiwan; Department of Surgery, Taipei Medical College Hospital, Taipei, Taiwan

**Keywords:** *TSG101* gene, transcripts, cervical cancer, endometrial cancer, tumour-suppressor gene

## Abstract

Carcinoma of the uterine cervix is a common malignancy among women that has been found to show loss of heterozygosity in the chromosome 11p. Recent studies have localized the *TSG101* gene in this region, and also demonstrated a high frequency of abnormalities of this gene in human breast cancer. To determine the role of the *TSG101* gene in the carcinogenesis of cervical and uterine carcinoma, 19 cases of cervical carcinoma and five cases of endometrial carcinoma, as well as nearby non-cancerous tissue from the same patients, and 16 blood samples from healthy persons as normal control were analysed by Southern blot analysis of genomic DNA, reverse transcription of the *TSG101* mRNA followed by PCR amplification and sequencing of the products. We found that abnormal transcripts of the *TSG101* gene were common both in cancerous or non-cancerous tissues of the uterus and cervix and in normal peripheral mononuclear cells. There was no genomic deletion or rearrangement in spite of the presence of abnormal transcripts, and no definite relationship between the abnormal transcripts and HPV infection was found. Although the frequency of abnormal transcripts was higher in cancerous than in non-cancerous tissue, normal peripheral mononuclear cells also had abnormal transcripts. Given these findings, the role of the *TSG101* gene as a tumour-suppressor gene should be re-evaluated. Because some aberrant transcripts could be found at the first PCR reaction, we suggest that the aberrant transcripts might be the result of imperfect minor splicesome products. © 1999 Cancer Research Campaign


*British Journal of Cancer* (1999) **79**(3/4), 445–450
© 1999 Cancer Research Campaign
Article no. bjoc.1998.0069

### Analysis of *TSG101* tumour susceptibility gene transcripts in cervical and endometrial cancers

**J-G Chang[1,2,3], T-H Su[4,5], H-J Wei[5], J-C Wang[1], Y-J Chen[6], C-P Chang[3] and C-J Jeng[4]**

[1]Department of Medical Research, Division of Molecular Medicine, Mackay Memorial Hospital, Taipei, Taiwan; [2]Department of Molecular Medicine, Taipei Municipal Jen-Ai Hospital, Taipei, Taiwan; [3]Department of Medical Research, China Medical College Hospital, Taichung, Taiwan; [4]Department of Obstetrics and Gynecology, Mackay Memorial Hospital, Taipei, Taiwan; [5]Department of Obstetrics and Gynecology, and [6]Department of Surgery, Taipei Medical College Hospital, Taipei, Taiwan

**Summary** Carcinoma of the uterine cervix is a common malignancy among women that has been found to show loss of heterozygosity in the chromosome 11p. Recent studies have localized the *TSG101* gene in this region, and also demonstrated a high frequency of abnormalities of this gene in human breast cancer. To determine the role of the *TSG101* gene in the carcinogenesis of cervical and uterine carcinoma, 19 cases of cervical carcinoma and five cases of endometrial carcinoma, as well as nearby non-cancerous tissue from the same patients, and 16 blood samples from healthy persons as normal control were analysed by Southern blot analysis of genomic DNA, reverse transcription of the *TSG101* mRNA followed by PCR amplification and sequencing of the products. We found that abnormal transcripts of the *TSG101* gene were common both in cancerous or non-cancerous tissues of the uterus and cervix and in normal peripheral mononuclear cells. There was no genomic deletion or rearrangement in spite of the presence of abnormal transcripts, and no definite relationship between the abnormal transcripts and HPV infection was found. Although the frequency of abnormal transcripts was higher in cancerous than in non-cancerous tissue, normal peripheral mononuclear cells also had abnormal transcripts. Given these findings, the role of the *TSG101* gene as a tumour-suppressor gene should be re-evaluated. Because some aberrant transcripts could be found at the first PCR reaction, we suggest that the aberrant transcripts might be the result of imperfect minor splicesome products.

**Keywords:** *TSG101* gene; transcripts; cervical cancer; endometrial cancer; tumour-suppressor gene

Carcinoma of the uterine cervix is a common malignancy affecting adult women worldwide, especially in developing countries. Epidemiological studies have suggested multiple risk factors in its aetiology (Wahi et al, 1969; Murthy et al, 1990; Howley, 1991). Inactivation or deletion of multiple tumour-related genes, which regulate normal cellular growth and suppression of abnormal cell proliferation, is recognized to be one of the major mechanisms of tumorigenesis. Mutations or deletions in the tumour-related genes are frequently accompanied by loss of the remaining allele or alleles (loss of heterozygosity, LOH), leading to homozygous inactivation of the gene(s) (Manchall, 1991; Hampton et al, 1994; Chridhar et al, 1996). Several studies have shown that LOH at specific chromosomal sites is frequently associated with the development of various cancers (Manchall, 1991). Some studies of cervical carcinoma have shown LOH on chromosome 11p, suggesting the presence of candidate tumour-suppressor genes on that chromosome (Busby-Earle, et al, 1993; Mitra et al, 1994). Recently, a gene, *TSG101*, was isolated and mapped to chromosome 11p15.1–15.2, a region proposed to contain tumour-suppressor genes that is mutated at high frequency in human breast cancer (Li and Cohen, 1996; Li et al, 1997). Because the LOH region in cervical carcinoma is close to the *TSG101* area, we analysed the mutation frequency of the *TSG101* gene in cervical and endometrial carcinomas, to investigate the role of *TSG101* in the development of both tumours. We also evaluated the relationship between the human papilloma infection and the transcripts of *TSG101*.

*Received 16 March 1998*
*Revised 15 June 1998*
*Accepted 13 July 1998*

*Correspondence to:* J-G Chang, Department of Medical Research, Division of Molecular Medicine, China Medical College Hospital, 2 Yuh Der Road, Taichung, Taiwan

### MATERIALS AND METHODS

### Specimens

Tissue samples from primary tumours and nearby non-tumorous tissue samples were obtained from five patients with endometrial cancer and 19 with cervical carcinoma who had been treated at Mackay Memorial Hospital (Taipei, Taiwan). All tissue samples were confirmed histologically. Clinically, two cases of endometrial cancer were stage Ib, and three were stage IIa. All these were adenocarcinoma. Of the cases of cervical cancer, eight were stage Ib, seven were IIa, two were IIb, and one case each was stage III or IV. All the cervical cancer cases were squamous cell carcinoma, 13 cases were well to moderately differentiated and six poorly differentiated. The proportion of tumour cells varied from 60% to 90%. The age of the patients ranged from 31 to 80 years old with a mean of 51 years. Peripheral mononuclear cells from 16 healthy persons were used as a normal control.

### DNA, RNA extraction and reverse transcription

Tumour specimens were frozen immediately after surgical resection and stored in liquid nitrogen until extraction of DNA or RNA.

This study had been presented as a poster at the 47th Annual Meeting of the American Society of Human Genetics. Drs JG Chang and TH Su have made equal contributions to this study.

**Table 1** Oligonucleotide primers for study of the *TSG101* gene

| Primers | Sequences | cDNA location[a] |
|---|---|---|
| First RT-PCR | | |
| P1u | 5′-AGCCCAGCAGCGGCTGACCCTCT-3′ | nt 35–57 |
| P1d | 5′-CTTATTCTGGGCACCTACTGAT-3′ | nt 1342–1321 |
| Second RT-PCR | | |
| P2u | 5′-CGGTGTCGGAGAGCCAGCTCAAG-3′ | nt 95–117 |
| P2d | 5′-CTCAACCTCCAGCTGGTATC-3′ | nt 1290–1271 |
| For sequencing | | |
| Ad | 5′-AGTAGCCATAGGCATATTTGG-3′ | nt 320–301 |
| Bu | 5′-GCCTTATAGAGGTAATACATAC-3′ | nt 273–294 |
| Bd | 5′-ATAGGATGCCGAAATAGGAC-3′ | nt 540–521 |
| Cu | 5′-CTTCCTTATCTACATGAATGG-3′ | nt 421–441 |
| Cd | 5′-CACCAGGTGGGTAAGGACAG-3′ | nt 670–651 |
| Du | 5′-GATACCCTCCCAATCCCAGTG-3′ | nt 620–640 |
| Dd | 5′-TTCGTTTCAAGGCATTGAGC-3′ | nt 865–846 |
| Eu | 5′-ATCTCTGCGGTCAGTGACA-3′ | nt 781–799 |
| Ed | 5′-AGGGAGCTGTGGGAATGATA-3′ | nt 1060–1041 |
| Fu | 5′-GGAAAAAATGGAAAATCAGTCTG | nt 996–1018 |

[a]According to nucleotide sequence of *TSG101* cDNA (GenBank accession number U82130). nt, nucleotide.

**Table 2** Aberrant transcripts of *TSG101* in cervical cancer

| Case | Age | Pathological finding | Abnormal transcript | | | |
|---|---|---|---|---|---|---|
| | | | Tumour | HPV | Non-tumour | HPV |
| 1 | 35 | SCC/II | IB$_1$,IA$_1$ | + | – | + |
| 3 | 56 | SCC/III | IB$_1$,IA$_4$ | – | ID,IA$_1$,IA$_4$ | – |
| 4 | 42 | SCC/III | IA$_1$,IB$_2$,IIB,IA$_3$ | – | – | – |
| 5 | 28 | SCC/II | IA$_3$,IA$_1$ | + | IA$_1$,IC,IID | + |
| 6 | 67 | SCC/III | IB$_2$,IIL | + | IID | + |
| 8 | 36 | SCC/II | IB$_2$,IIB,IA$_3$ | + | IB$_2$,IA$_1$ | + |
| 9 | 31 | SCC/II | IB$_1$,IB$_2$ | + | IIH | + |
| 13 | 59 | SCC/III | IB$_2$,IA$_1$ | + | IA$_1$ | + |
| 14 | 36 | SCC/II | IE | + | IIM | + |
| 15 | 73 | SCC/II | IB$_2$,IIK,IA$_1$ | – | II I,IID | – |
| 16 | 63 | SCC/II | IB$_2$ | + | – | – |
| 17 | 67 | SCC/II | IA$_1$,IA$_4$ | – | – | – |
| 18 | 50 | SCC/III | IIM | + | IA$_4$ | + |
| 19 | 48 | SCC/II | IID,IA$_1$,IIA | + | IIF,IA$_1$ | + |
| 20 | 48 | SCC/III | IE,IB$_2$,IA,IIF | + | IE,IA$_1$ | + |
| 21 | 35 | SCC/II | IB$_2$,IID | + | IIF,IIE,IB$_1$,IIA,IA$_1$,IA$_4$ | + |
| 22 | 56 | SCC/III | IID,IE,IB$_2$,IB$_1$,IA$_1$,IA$_4$, | + | IE,IB$_2$,IIA, IA$_1$,IA$_4$,IIJ | + |
| 23 | 48 | SCC/II | IB$_2$,IB$_1$,IIK,IA$_1$ | + | IA$_2$,IIM | + |
| 24 | 55 | SCC/II | IE,IB$_2$,IA,IA$_4$ | + | – | + |

IA$_1$, nt 132–729 deletion; IA$_2$, nt 132–637 deletion; IA$_3$, nt 133–933 deletion; IA$_4$, nt 133–447 deletion; IB$_1$, nt 154–1054 deletion; IB$_2$, nt 154–1070 deletion; IC, nt 166–1113 deletion; ID, nt 172–1194 deletion; IE, nt 230–1212 deletion; IF, nt 284–638 deletion; IIA, nt 120–873 deletion; IIB, nt 125–1007 deletion; IIC, nt 145–1072 deletion; IID, nt 150–1157 deletion; IIE, nt 151–1102 deletion; IIF, nt 160–1182 deletion; IIG, nt 184–1259 deletion; IIH, nt 200–959 deletion; II I, nt 250–1221 deletion, IIJ, nt 262–1160 deletion; IIK, nt 335–1152 deletion; IIL, nt 399–1178 deletion; IIM, nt 443–830 deletion; IIN, nt 687–1112 deletion; nt, nucleotide; SCC, squamous cell carcinoma.

The DNA extraction was performed as previously described (Lin et al, 1993). Total RNA was purified from cancerous and nearby non-cancerous tissues and peripheral mononuclear cells using a commercial kit (RNAzolTMB, Biotecx laboratories, Houston, TX, USA). RNA was stored as a pellet in ethanol or solubilized in RNAase-free water and kept at –70°C. Reverse transcription was performed as described previously (Chen et al, 1997).

### Reverse transcription polymerase chain reaction (RT-PCR), long PCR, Southern blot analysis and sequencing

The primers used for RT-PCR analysis of the *TSG101* gene are shown in Table 1. The PCR conditions were as described, but with some modifications (Chen et al, 1997). Briefly, 1 µl of cDNA was used for the first PCR amplification in a volume of 100 µl

 *© Cancer Research Campaign 1999*

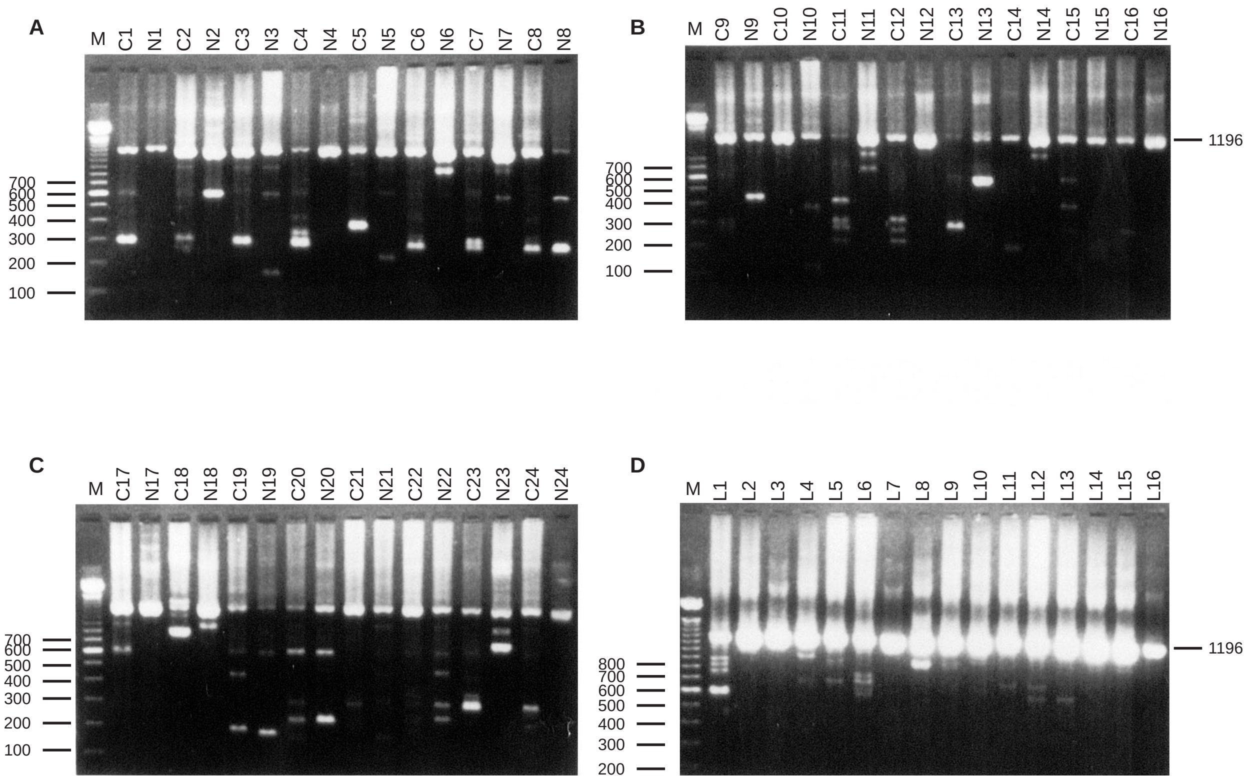

**Figure 1** Nested RT-PCR amplification of *TSG101* from cervical and endometrial cancer tissues (**A**, **B**, **C**). Cases 2, 7, 10, 11 and 12 were from cases of endometrial cancer. The rest were cervical cancer. C, cancerous tissue. N, non-cancerous tissue. The results of peripheral mononuclear cells are shown in **D**. L, peripheral mononuclear cells. The 1196-bp band is the normal *TSG101* gene transcript. The smaller bands are aberrant transcripts containing 100 ng each of primers P1u and P1d, 50 μM of each dNTP, 1 × PCR buffer, and 2.5 units polymerase. PCR was performed for 35 cycles of 1.5 min at 94°C for denaturation, 1.5 min at 56°C for annealing, and 2.5 min at 72°C for extension, with a final extension of 5 min at 72°C using a Perkin-Elmer Cetus PCR Thermocycler. Of the first PCR products, 1–10 μl were subjected to a second round of PCR amplification using nested primers P2u and P2d for 35 cycles under the above conditions. The PCR products were electrophoresed and analysed using agarose gel. For long PCR, amplification of genomic DNA, the primers and PCR conditions were identical to the study of Li et al, 1997). We also used the primer located near the breakpoint regions to amplify the breakpoints (Table 1). The PCR products of nested RT-PCR and long PCR were subjected to Southern blot analysis as follows: the DNA on agarose gel was Southern transferred to a nylon membrane. The membrane was hybridized with a random-primer $^{32}$P-labelled probe (DECA primer II DNA labelling kit, Ambion, Austin, TX, USA) which corresponded to the cDNA of *TSG101*. All positively labelled fragments were subjected to isolation and sequencing analysis. Isolation was achieved by electrophoresis in a 3% low melting agarose gel; the fragments were excised after separation. They were then purified using a commercial kit (Qiaex II gel extraction kit, Qiagen, Chatsworth, CA, USA), and the purified fragments were sequenced by the cycling sequencing method using an ABI 310 automatic sequencer. The sequencing primers are also shown in the Table 1.

### Southern blot analysis of genomic DNA

Genomic DNA (5–10 μg) was digested with restriction enzymes *Eco*RI, *Hin*dIII and *Sac*I according the manufacturer's instructions, electrophoresed in 0.8% agarose gel, Southern transferred to Hybond H+ membrane, fixed by UV cross-linking, and the membrane was hybridized with a random-primer $^{32}$P-labelled cDNA probe as mentioned above. Loss or gain of restriction fragments was assessed by visual comparison of hybridization signals after developing the exposed radiographic film.

### Human papillomavirus (HPV) analysis

The HPV DNA sequence in tumour samples was amplified using the pU-1M/PU-2R primer pair to amplify malignant HPV DNA (HPV 16, 18, 31, 33, 52b and 58); benign HPV DNA (HPV 6 and 11) was amplified using the pU-31B/pU-2R primer pair. The primers and PCR procedures were identical to those used in the study of Fujinaga et al (1991).

### RESULTS

### RT-PCR amplification and sequence analysis

RT-PCR analysis and sequencing of the PCR fragments revealed frequent expression of both the wild-type *TSG101* and intragenic deletions in cancerous and non-cancerous tissues. The results of

© Cancer Research Campaign 1999 

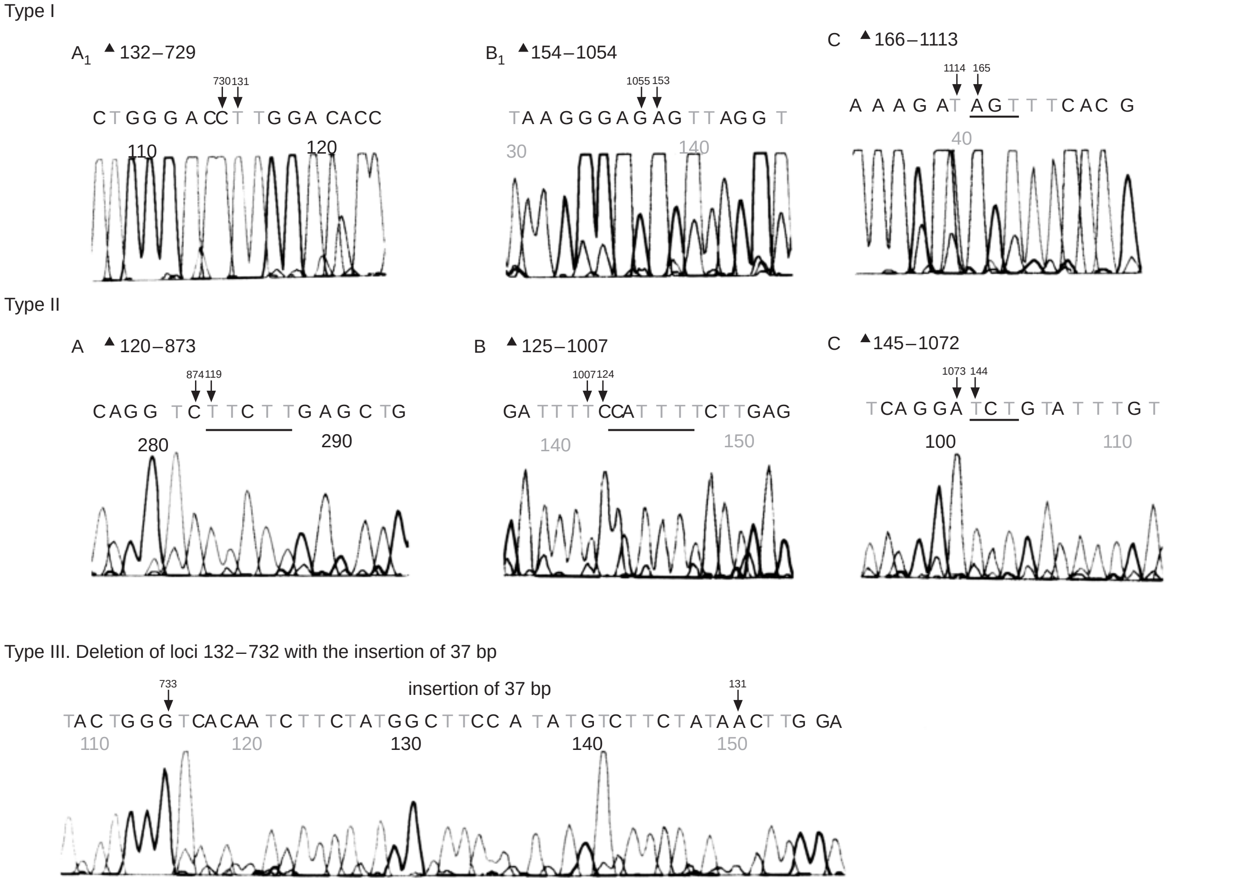

**Figure 2** Sequence at *TSG101* cDNA deletion junction in representative cases of cancerous and non-cancerous tissues and normal peripheral mononuclear cells. Numbers above each figure indicate abnormal junction between deletions abnormal transcript analysis are shown in Figures 1(A–C) and 2. In addition to the wild-type transcript, most cancerous tissues (100% of cervical carcinomas and 80% of endometrial carcinomas) as well as most of the non-cancerous tissues (73.7% of nearby cervical tissues and 80% of paired endometrial tissues) demonstrated one or more aberrant *TSG101* transcripts (Figure 1). The aberrant band sizes were diverse (120–881 bp) with a range of one to six bands in any one specimen. Upon sequencing, we found that the aberrant bands were due to intragenic deletions, including 25 different types of deletion and deletion sizes range from 387 bp to 1075 bp. According to region, at least three major categories of deletion could be found. Group 1 was similar to those found by Lee and Feinberg (1997), and may be due to relaxation of the splicing mechanism (Figure 3, type 1). Group 2 had a common homologous sequence between the donor and acceptor area (Figure 3, type II). Group 3 had a deletion and an insertion (Figure 3, type III). Cancerous and non-cancerous tissues did not differ in the types of deletions found. The sequencing results of aberrant bands are shown in Table 2 and Figure 2.

### Genomic long PCR and genomic Southern blot analysis

For further analysis of the breakpoint, we followed long PCR method as described by Li et al (1997). At the expected breakpoint site on the gel, no PCR products could be found with *TSG101*

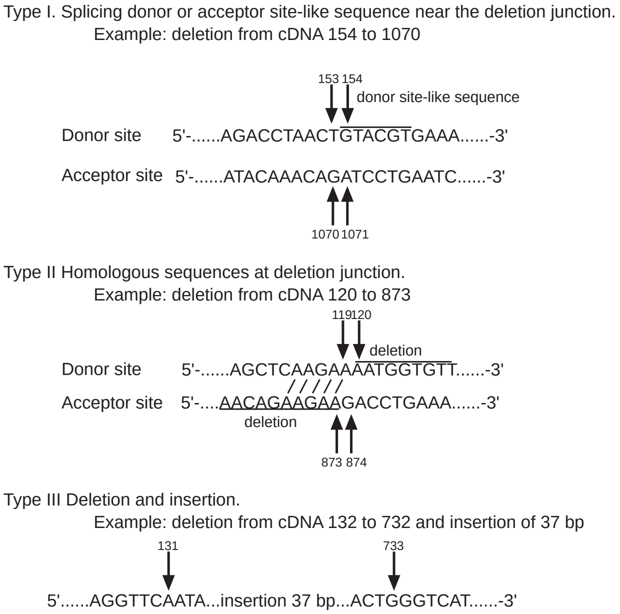

**Figure 3** Sequencing analysis of aberrant RNA splicing. There are three types of aberrant transcripts. Type I has a splicing donor or acceptor site sequence at the deletion junction region. Type II has a homologous sequence between the deletion junction region. Type III has both deletion and insertion

*© Cancer Research Campaign 1999*

**Table 3** Aberrant transcripts of *TSG101* in endometrial cancer

| Case | Age | Pathological finding | Abnormal transcript | |
|---|---|---|---|---|
| | | | Tumour | Non-tumour |
| 2 | 68 | Adenocarcinoma | $IB_1$,$IA_4$,$IA_1$ | $IA_1$ |
| 7 | 36 | Adenocarcinoma | $IB_1$,$IB_2$,$IA_1$,$IA_4$,$IA_3$ | III,IID |
| 10 | 57 | Adenocarcinoma | | IIG,IIK |
| 11 | 80 | Adenocarcinoma | II I,IB,$IB_2$,IIL | $IA_4$,$IA_2$ |
| 12 | 55 | Adenocarcinoma | II I,$IB_1$,IIC | – |

**Table 4** Aberrant transcripts of *TSG101* in peripheral mononuclear cells of normal controls

| Case no. | Abnormal transcript |
|---|---|
| $L_1$ | $IA_1$,IIM,$IA_4$,IIN |
| $L_2$,$L_3$,$L_7$,$L_{10}$,$L_{14}$,$L_{15}$,$L_{16}$ | – |
| $L_4$ | $IA_2$,$IA_4$ |
| $L_5$ | $IA_2$,IF |
| $L_6$ | $IA_1$,IIN,$IA_2$,III |
| $L_8$ | IF |
| $L_9$ | IF |
| $L_{11}$ | $IA_2$ |
| $L_{12}$ | $IA_1$,$IA_2$ |
| $L_{13}$ | $IA_1$ |

cDNA probe detection (data not shown). We also used primers much closer to the intragenic deletion region to amplify the breakpoint. However, we were unable to amplify the breakpoint region. To further demonstrate gross deletion of the *TSG101* gene, we used Southern blot analysis of genomic DNA to detect deletion fragments. There were no gross deletions despite the presence of aberrant transcripts (data not shown). The lack of any altered DNA fragment on direct genomic Southern blots and long PCR analysis in any of the 19 cervical and five endometrial cancerous or non-cancerous samples, including those with aberrant transcripts, indicated that the aberrant transcripts were not caused by intragenic deletion of sufficient size to explain them.

### HPV analysis

None of the patients in this study with endometrial cancer had HPV infection. Of 19 patients with cervical cancers (73.7%), 14 had detectable HPV DNA, of which 11 were HPV 16, two HPV 18, one HPV16+31, one HPV 31, and one HPV 58. Of 18 non-cancerous tissues, ten demonstrated HPV infection, of which five tissues were infected by HPV 16, one by HPV 18, and four by HPV 31. However, no definite relationship between HPV infection and the abnormal *TSG101* transcript was found (Table 2).

### Aberrant transcripts of TSG101in normal peripheral mononuclear cells

RT-PCR analysis and sequencing of the PCR fragments in normal peripheral mononuclear cells revealed frequent expression of the wild-type and intragenic deletion of *TSG101* transcripts. The results of abnormal transcript analysis are shown in the Figure 1D and Table 2. In addition to the wild-type transcript, 9 (56.25%) out of 16 demonstrated one to four aberrant *TSG101* transcripts (Figure 1D).

### DISCUSSION

Li et al (1997), based on the presence of truncated transcripts found by RT-PCR and Southern hybridization of PCR products of genomic DNA, reported the presence of large intragenic deletions of the *TSG101* gene in 7 out of 15 primary human breast cancers, but none in matched normal breast tissues. Lee et al (1997) used a similar approach to analyse 72 human breast cancer and matched non-cancerous tissues. They found that the aberrant transcripts were common in both cancerous and non-cancerous tissues; no intragenic deletions were found in their cases using long PCR or Southern blot analysis of genomic DNA. Steiner et al (1997) performed Southern blot analysis of genomic DNA from human breast cancer cell lines and normal peripheral blood. They were unable to find intragenic deletion of *TSG101* gene. In this study, we used a similar approach to investigate the role of *TSG101* in the development of cervical and endometrial cancers. We also found that the aberrant transcripts were common in both cancerous and non-cancerous tissues, and that there were no intragenic deletions, point mutations or small deletions/insertions on genomic Southern blot analysis and whole cDNA sequencing analysis. Our results were, thus, similar to the studies of Lee et al (1997) and Steiner et al (1997).

Lee et al (1997) suggested that the relaxation of RNA splicing may, in general, contribute to abnormal gene expression. We found a new type of aberrant transcript that had a homologous sequence between donor and acceptor sites. We suggest that this type of aberrant transcript may be related to RNA secondary structure. We found some aberrant transcripts could be found at the first PCR reaction, such as deletion from nucleotide 154 to 1054 or from nucleotide 154 to 1070, which has highly homologous splicing and acceptor site-like sequence at the deletion junction. From this point of view, we suggest that the aberrant transcripts might be the result of imperfect minor splicesome products.

The aberrant splicing of *TSG101* gene appears to be identified more frequently in cancerous than in non-cancerous tissues (100% vs. 72.5%), but it also appears in peripheral mononuclear cells of normal persons. For these reasons, the role of *TSG101* gene as a tumour-suppressor gene cannot yet be confirmed and requires much more careful evaluation.

### ACKNOWLEDGEMENTS

This study was in part supported by a grant from NSC, Taiwan, ROC (NSC 88-2314-B-195-014), and a grant from Mackay Memorial Hospital (MMH8714).

### REFERENCES

Busby-Earle RMC, Steel CM and Bird CC (1993) Cervical carcinoma: low frequency of allele loss at loci implicated in other common malignancies. *Br J Cancer* **67**: 71–75

Chen YJ, Chang JG, Chih LS, Chen PH, Endo M, Whang-Peng J and Chen YMA (1997) Frequent detection of aberrant RNA transcripts of the CDKN2 gene in human gastric adenocarcinoma. *Int J Cancer* **71**: 350–354

Chridhar R, Sridhar V, Wang X, Paradee W, Dugen M, Sarker F, Wike, Glover TW, Vaitkevicius VK and Smith DI (1996) Frequent breakpoints in the 3p14.2 fragile site, FRA3B, in pancreatic tumors. *Cancer Res* **56**: 4347–4350

Fujinaga Y, Shimada M, Okazawa LK, Fukushima M, Kato I and Fujinagea K (1991) Simultaneous detection and typing of genital human papillomavirus DNA typing using the polymerase chain reaction. *J Gen Virol* **72**: 1039–1044

Hampton GM, Penny LA, Baergen RN, Larson A, Brewen C, Liao S, Busby-Earle RMC, Williams AWR, Steel CM, Bird CC, Stanbridge EJ and Evans GA (1994) Loss of heterozygosity in cervical carcinoma: subchromosomal localization of a putative tumor-suppressor gene to chromosome 11q22–q24. *Proc Natl Acad Sci USA* **91**: 6953–6957

Howley PM (1991) Role of human papillomaviruses in human cancer. *Cancer Res* **51** (suppl.): 5019–5022

Lee MP and Feinberg AP (1997) Aberrant splicing but not mutations of *TSG101* in human breast cancer. *Cancer Res* **57**: 3131–3134

Li L and Cohen SN (1996) *TSG101*: a novel tumor susceptibility gene isolated by controlled homozygous functional knockout of allelic loci in mammalian cells. *Cell* **85**: 319–329

Li L, Li X, Francke U and Cohen SN (1997) The *TSG101* tumor susceptibility gene is located in chromosome 11 band p15 and is mutated in human breast cancer. *Cell* **88**: 143–153

Lin SY, Chen PH, Wang CK, Liu JD, Siauw CP, Chen YJ, Yang MJ, Liu MH, Chen TC and Chang JG (1993) Mutation analysis of K-*ras* oncogenes in gastroenterologic cancers by the amplified created restriction sites method. *Am J Clin Pathol* **100**: 686–689

Manchall CJ (1991) Tumor suppressor genes. *Cell* **64**: 313–326

Mitra AB, Morty VVVS, Li Rong G, Mahendra Pratop, Luthra UK and Chaganti RSK (1994) Allelotype analysis of cervical carcinoma. *Cancer Res* **54**: 4481–4487

Murthy NS, Sehgal A, Satyonarayana L, Das D, Singh V, Das BC, Gupta MM, Mitra AB and Luthra UK (1990) Risk factors related to biological behaviour of precancerous lesions of the uterine cervix. *Br J Cancer* **61**: 732–736

Steiner P, Barnes DM, Haris WH and Weinberg RA (1997) Absence of rearrangements in the tumor susceptibility gene *TSG101* in human breast cancer. *Nature Genet* **16**: 332–333

Wahi PH, Mali S and Luthra UK (1969) Factors influencing cancer of the cervix in north India. *Cancer* **23**: 1221–1226

© Cancer Research Campaign 1999
